# Recombinant EGFL7 Mitigated Pressure Overload-Induced Cardiac Remodeling by Blocking PI3K γ /AKT/ NFκB Signaling in Macrophages

**DOI:** 10.3389/fphar.2022.858118

**Published:** 2022-05-26

**Authors:** Lei Li, Ying Zhao, Ying Hu, Xiaohui Wang, Qun Jin, Ying Zhao

**Affiliations:** ^1^ Department of Cardiology, Shandong Provincial Hospital, Cheeloo College of Medicine, Shandong University, Jinan, China; ^2^ The Key Laboratory of Cardiovascular Remodeling and Function Research, Chinese Ministry of Education, Chinese National Health Commission and Chinese Academy of Medical Sciences, The State and Shandong Province Joint Key Laboratory of Translational Cardiovascular Medicine, Department of Cardiology, Qilu Hospital, Cheeloo College of Medicine, Shandong University, Jinan, China; ^3^ Department of Geriatrics, 960 Hospital of PLA(The General Hospital of Jinan Command), Jinan, China; ^4^ Department of Cardiology, Liao Cheng People’s Hospital, Liao Cheng, China; ^5^ Department of Medical Records, Heze Municipal Hospital, Heze, China

**Keywords:** macrophage, EGFL7, adhesion molecule, interaction, remodeling, heart failure

## Abstract

Inflammation and endothelial dysfunction play an essential role in heart failure (HF). Epidermal growth factor-like protein 7 (EGFL7) is upregulated during pathological hypoxia and exerts a protective role. However, it is unclear whether there is a link between abnormal EGFL7 expression and inflammation in overload stress-induced heart failure. Our results showed that EGFL7 transiently increased during the early 4 weeks of TAC and in hypertensive patients without heart failure. However, it decreased to the basal line in the heart tissue 8 weeks post-transverse aortic constriction (TAC) or hypertensive patients with heart failure. Knockdown of EGFL7 with siRNA *in vivo* accelerated cardiac dysfunction, fibrosis, and macrophage infiltration 4 weeks after TAC. Deletion of macrophages in siRNA-EGFL7-TAC mice rescued that pathological phenotype. *In vitro* research revealed the mechanism. PI3K γ /AKT/N FκB signaling in macrophages was activated by the supernatant from endothelial cells stimulated by siRNA-EGFL7+phenylephrine. More macrophages adhered to endothelial cells, but pretreatment of macrophages with PI3Kγ inhibitors decreased the adhesion of macrophages to endothelial cells. Ultimately, treatment with recombinant rmEGFL7 rescued cardiac dysfunction and macrophage infiltration in siRNA-EGFL7-TAC mice. In conclusion, EGFL7 is a potential inhibitor of macrophage adhesion to mouse aortic endothelial cells. The downregulation of EGFL7 combined with increased macrophage infiltration further promoted cardiac dysfunction under pressure overload stress. Mechanistically, EGFL7 reduced endothelial cell adhesion molecule expression and inhibited the PI3K γ /AKT/NF κ B signaling pathway in macrophages.

## Introduction

Heart failure (HF) is a complex clinical syndrome with high morbidity and mortality and exerts a staggering social burden. Traditional drugs have brought obvious beneficial outcomes in the past several decades, especially in HF with a reduced ejection fraction (HFrEF). Nevertheless, such treatments gain little practical response in HF with a preserved ejection fraction HFpEF (Dick and Epelman, 2016). Increased proinflammatory cytokines and endothelial dysfunction are common in both HFrEF and HFpEF and are inversely related to prognosis (Edelmann et al., 2015; Mann, 2015). Inflammation and endothelial dysfunction interact with each other and form vicious cycles. Although some anti-inflammatory monoclonal antibodies, such as canakinumab and natalizumab, significantly reduce lesions, they also compromise physiological protection against infection (Edelmann et al., 2015; Mann, 2015). Therefore, great effort is needed to explore the pathophysiological mechanism in pressure overload-induced heart failure.

Epidermal growth factor-like protein 7 (EGFL7) is a highly conserved, secreted extracellular matrix binding factor uniquely expressed by endothelial cells (Nichol and Stuhlmann, 2012). EGFL7 is upregulated during physiological angiogenesis in parallel with proliferated ECs and tissues. Under vascular damage or hypoxic environments, EGFL7 is temporarily upregulated (Parker et al., 2004). Suzanne Delfortrie et al. (2011) showed that EGFL7 plays a regulatory role in the tumor immune microenvironment and inhibits immune cell infiltration (Delfortrie et al., 2011).

The link between EGFL7 and inflammation in pressure overload-induced heart failure has not been studied. Pharmacological and genetic manipulation of interfering chemokine subsets of PI3K γ  has gained potential therapeutic implications in multiple animal models, including heart failure (Oudit et al., 2003; Patrucco et al., 2004; Vecchione et al., 2005; Rückle et al., 2006). As inflammation plays a vital role in heart failure, we hypothesized that EGFL7 is protective in pressure overload-induced hypertrophy and the heart failure model TAC. We demonstrated the protective role of EGFL7 in mediating endothelium-macrophage interactions by reducing adhesion molecules in ECs and blocking the PI3K γ /AKT/ NFκ B signaling pathway in macrophages.

## Methods

### Human Serum Samples

We obtained serum samples from controls, patients with hypertension, or hypertension and heart failure from 960 Hospital of PLA. The research was approved by the institutional ethics committee of 960 Hospital of PLA (2016. No.56). All participants provided informed written consent.

The following experiments are described in the Supplementary Material, material and methods section after discussion: reagents, primers, cell culture, construction of the siRNA-EGFL7 sequence, recombinant mouse EGFL7, TAC surgery in wild-type and siRNA-EGFL7 (2′Ome) and pharmacological treatment, echocardiography, histological analysis, including immunohistochemical (IHC)/Masson/HE staining, western blotting, Q-RT–PCR, adhesion analysis, TUNEL, and ELISA.

### Statistical Analysis

Results are presented as the mean ± SD. One-way or two-way ANOVA followed by Tukey’s test or Bonferroni’s test was used to compare>2 groups. All statistics were analyzed by GraphPad Prism 9. *p* values < 0.05 were regarded as statistically significant.

## Results

### Epidermal Growth Factor-Like Protein 7 is Differentially Expressed in Hypertensive Patients With or Without Heart Failure

**FIGURE 1:**
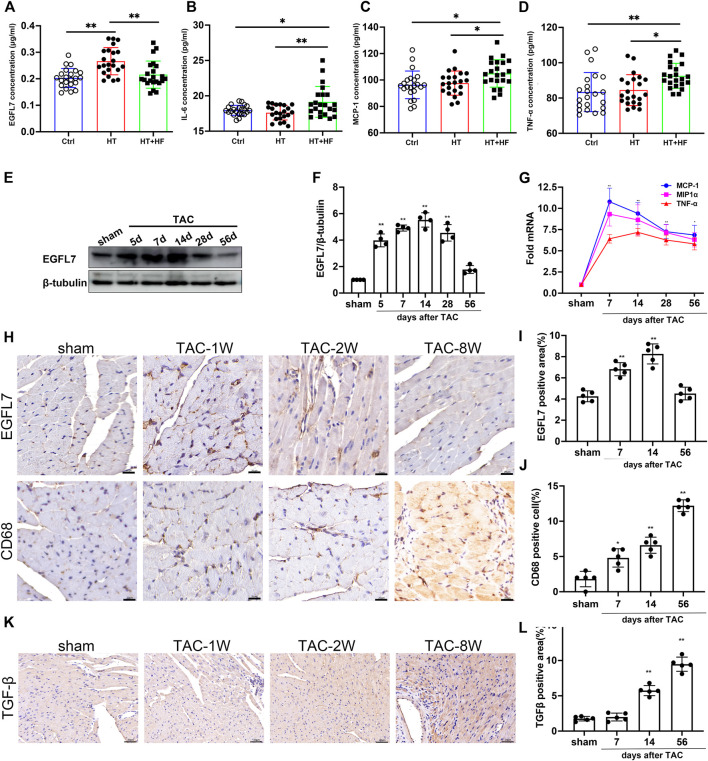
EGFL7 is differently expressed in hypertrophic heart with or without heart failure and TAC-induced heart failure. Expression of EGFL7 **(A)** and IL-6 **(B)**, MCP-1 **(C)**, TNF-α **(D)** in serum of patients from healthy control, hypertension, and hypertension with heart failure. Data are presented as mean ± SD (*n* = 22 for each group, *p < 0.05, **p < 0.01 by one-way ANOVA followed mean ± SD by Tukey’s test). **(E)** C57BL/6J mice were subjected to either sham (*n* = 6) or TAC operation and observed after 5, 7, 14, 28, 56 days (*n* = 6 respectively). Representative immunoblots of EGFL7 expression in cardiac tissue among multiple time points post TAC. **(F)** Quantitative analysis expression of EGFL7. The values were normalized to β-tubulin. (**p < 0.01 vs. sham by one-way ANOVA followed by Tukey’s test). **(G)** Guantitative analysis of inflammatory cytokines mRNA of MCP-1, MIP1α, TNF-α in heart tissue of TAC, values were normalized to GAPDH. (**p < 0.01 vs. sham by one-way ANOVA followed by Tukey’s test). **(H)** Representative immunohistochemical staining of EGFL7, CD68. Scale bar = 20 μm **(I, J)** Quantitative analysis of EGFL7, CD68 expression. Data were qualified from 6 images per sample. Quantification of positive area % was calculated using ImageJ software. (*p < 0.05, **p < 0.01 with sham by one-way ANOVA followed by Tukey’s test). **(K, L)** Representative immunohistochemical staining of TGF-β and quantitative analysis. Quantification of positive area % was calculated using ImageJ software (**p < 0.01 with sham by one-way ANOVA followed by Tukey’s test). Scale bar = 20 μm. All data represent the mean ± SD from at least 4 independent experiments.

Few previous studies have shown the relevance of EGFL7 in hypertrophy and heart failure. To explore the potential role of EGFL7, we first detected the expression of EGFL7. We collected serum samples from healthy controls, hypertensive (HT) patients, and hypertensive patients with heart failure (HT + HF). Our results showed upregulation of EGFL7 in the HT group compared with the control. However, EGFL7 expression decreased in the HT + HF group (Figure 1A). Other inflammatory cytokines, including IL-6, MCP-1, TNF-α were expressed differently. They were significantly upregulated in the HT + HF group compared to the others (Figures 1B–D).

### Epidermal Growth Factor-Like Protein 7 is Transiently Increased in Cardiac Tissue During the Early Phase of Heart Failure but Is Gradually Downregulated During the Chronic Phase of Transverse Aortic Constriction-Induced Heart Failure

To further confirm whether EGFL7 downregulation was related to cardiac dysfunction, C57BL/6J mice were subjected to transverse aortic constriction (TAC). EGFL7 expression was upregulated on the 5th day after TAC, peaked until the 14th day, and gradually decreased. At 4 weeks after TAC, there remained a statistical difference. However, EGFL7 expression decreased to normal on the 56th day after TAC (Figures 1E,F)**.**

Immunohistochemical analysis also confirmed the trend of EGFL7 expression (Figures 1H,I). The mRNA expression levels of inflammatory cytokines, including MCP-1, MIP1α, and TNF-α, were upregulated approximately 5- to 10-fold on the 7th day after TAC. They were maintained at a high level until 56 days post-TAC compared with the sham group (Figure 1G). Meanwhile, we detected infiltration of CD68^+^ macrophages. More macrophages were observed in the 8-week TAC group (Figures 1H,J). Immunohistochemical analysis indicated that TGF-β increased on the 14th day after TAC and continued to rise on the 56th day (Figures 1K,L).

### Epidermal Growth Factor-Like Protein 7-KD is Associated With Deterioration of Cardiac Remodeling in Transverse Aortic Constriction

**FIGURE 2:**
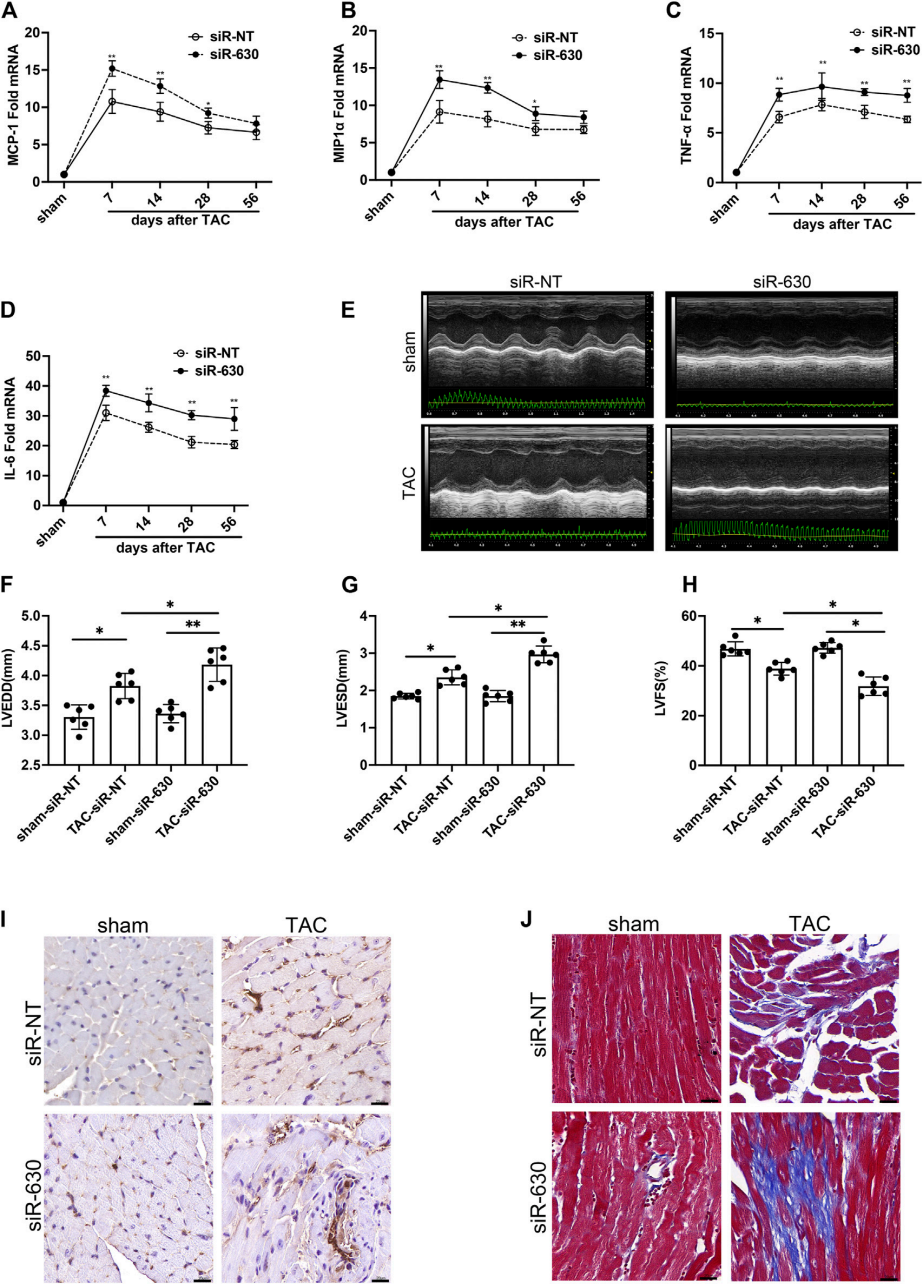
EGFL7-KD is associated with deterioration of cardiac remodeling in TAC. C57BL/6J mice were subjected to either sham or TAC operation, then 2′Ome modified miRNA-630 (2 umol/L, twice a week) or siRNA-NT was intraperitoneally injected the second-day post-TAC. Echocardiograms and morphology changes were observed 4 weeks after TAC (*n* = 6 in each group). **(A)** expression and quantitative analysis of inflammatory cytokines mRNA of MCP-1 and MIP1α **(B)**, TNF-α **(C)**, IL-6 **(D)** (*p < 0.05, **p < 0.01 vs. relative siRNA-NT by two-way ANOVA followed by Bonferroni’s test). (EFGH) representative images of echocardiograms and quantitative analysis of left ventricular end-diastolic diameter (LVEDD), left ventricle end-systolic diameter (LVESD), left ventricle fractional shortening (LVFS) between different groups (*p < 0.05, **p < 0.01 by one-way ANOVA followed by Tukey’s test). **(I)** Representative immunohistochemical staining of CD68 indicated infiltration of macrophages. scale bar = 20 μm. **(J)** Representative Masson staining indicated interstitial fibrosis was increased in siRNA-630-TAC. Scale bar = 20 μm. All data represent the mean ± SD from at least 4 independent experiments.

These results indicated different expressions during the early and late stages of heart failure. Elevated EGFL7 expression in the first 4 weeks when the heart remained in compensatory stage, paralleled with mild macrophage infiltration and fibrosis. In decompensated heart failure, EGFL7 decreased to baseline. Meanwhile, infiltration of macrophages increased and upregulated inflammatory cytokine expression. We want to explore the role EGFL7 plays during heart failure progression. So 4 weeks after TAC was the time point of intervention. The next step was to determine what role EGFL7 plays during heart failure progression. siRNA fragments of EGFL7 were constructed. After screening, siRNA630 achieved at least 85% reduction efficiency in EGFL7 protein expression (Supplementary Figures S1A,B). 2′-OMe-modified siRNA-630 was constructed and intraperitoneally injected. Western blotting showed an approximately 80% loss of EGFL7 in heart tissue. There were no baseline differences in cardiac function or morphology in mice that received siRNA-630 and siRNA-NT (Supplementary Table S3). Mice injected with siRNA-630, or siRNA-NT underwent TAC surgery. mRNA of MCP-1, MIP1α, IL-6, and TNF-α significantly elevated the 7th-day post-TAC, with approximately 5- to 30-fold increases in the siRNA-NT-TAC group versus the sham group. Then, they maintained high levels until 56 days post-TAC. siRNA-630-TAC caused an even more significant response in these genes (Figures 2A–D)**.** Left ventricular dilatation with contractive dysfunction indicated by LVEDD, LVESD, LVFS in the siRNA-630-TAC group was significantly more severe than in the siRNA-NT-TAC group 4 weeks after TAC (Figures 2E–H). Heart weight, lung weight, liver weight, LVPWd (diastolic left ventricle posterior wall thickness) showed significant differences (Supplementary Table S4). Immunohistochemical staining and quantitative analysis showed more infiltration of CD68^+^ macrophages in the siRNA-630-TAC group (Figure 2I, Supplementary Figure S1C) compared with the siRNA-NT-TAC group. Interstitial fibrosis indicated by the Masson staining showed a significant increase in siRNA-630-TAC (Figure 2J, Supplementary Figure S1D)**.**

### Macrophage Infiltration Induces Inflammation and Cardiac Remodeling in Epidermal Growth Factor-Like Protein 7 KD Mice

**FIGURE 3:**
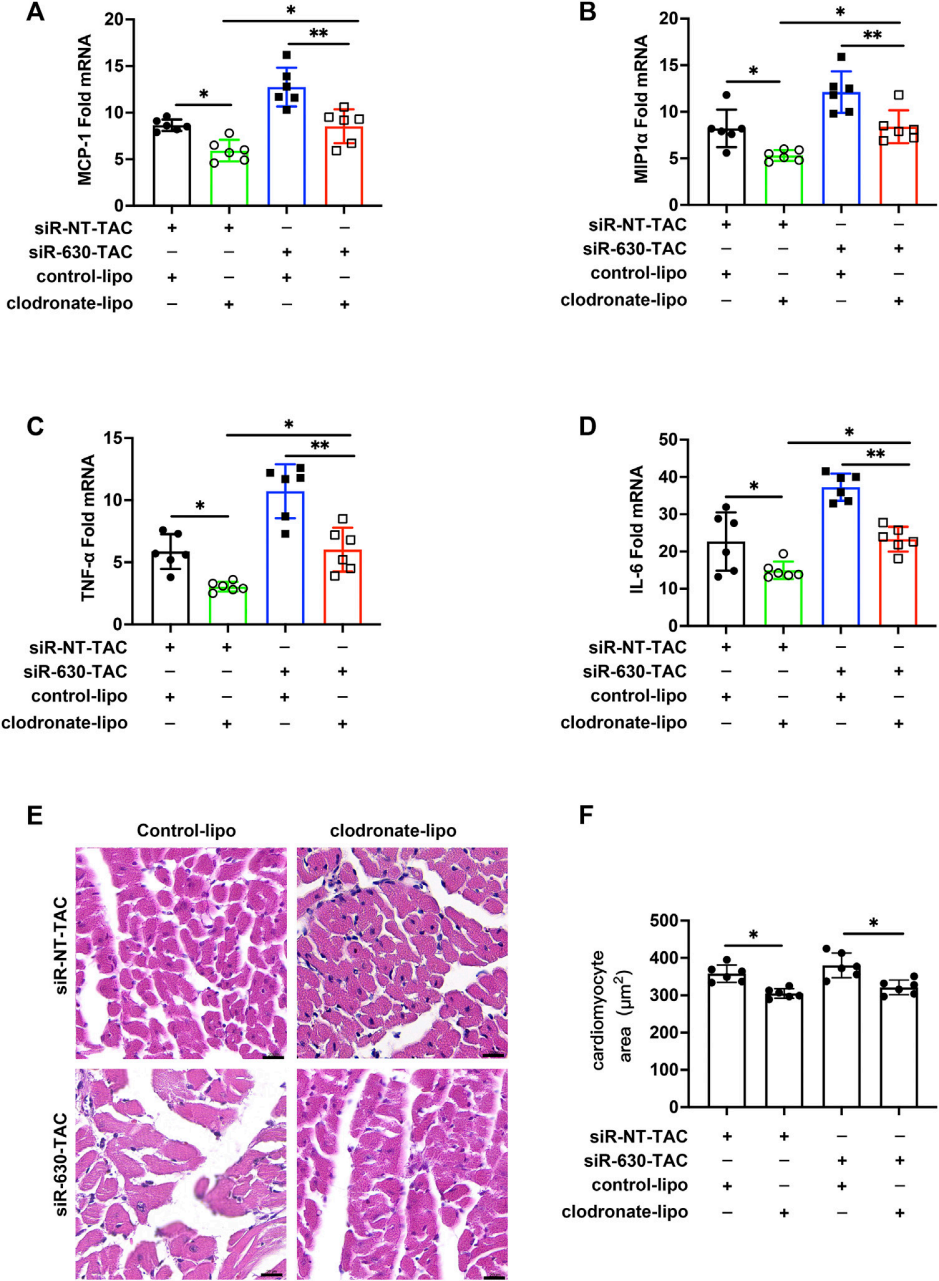
Depletion of macrophages mitigated inflammation and cardiac remodeling in EGFL7 KD mice. C57BL/6J mice were subjected to either sham or TAC operation, then clodronate liposomes or clodronate control was tail vein injected every other day from the second-day post-TAC for 4 weeks. siRNA-NT or siRNA-630 was intraperitoneal injection twice a week from the second-day post-TAC for 4 weeks. Morphological measure observed at 4 weeks. (*n* = 6 in each group). **(A)** Expression of inflammatory cytokine mRNA including MCP-1, MIP1α **(B)**, TNF-α **(C)**, and IL-6 **(D)** in heart tissue of TAC mice treated with clodronate liposomes or control (*p < 0.05, **p < 0.01 by one-way ANOVA followed by Tukey’s test). **(E, F)** Representative hematoxylin-eosin staining indicated cardiomyocytes cross-sectional area. The quantitative analysis of the cardiomyocyte area was calculated using ImageJ software (*p < 0.05 by one-way ANOVA followed by Tukey’s test). Scale bar = 20 μm. All data represent the mean ± SD from at least 4 independent experiments.

In siRNA-630-TAC mice, more macrophages infiltrated; thus, we wanted to explore what role macrophages play in the pathological process. Clodronate liposomes were used to deplete macrophages through tail vein injection (Xia et al., 2019). The depletion efficiency of clodronate liposomes was confirmed by measuring spleen macrophages of mice 2 days after injection (Supplementary Figure S2). After the TAC procedure, mRNA of MCP-1, MIP1α, TNF-α, and IL-6 downregulated in the clodronate liposome group (Figures 3A–D). Clodronate liposomes also decreased cardiomyocyte area (Figures 3E,F). Masson staining and quantitative indicated reduced interstitial fibrosis compared with the siRNA-NT-TAC group (Supplementary Figure S3).

### Epidermal Growth Factor-Like Protein 7 Represses Adhesion Molecule Expression in Endothelial Cells

**FIGURE 4:**
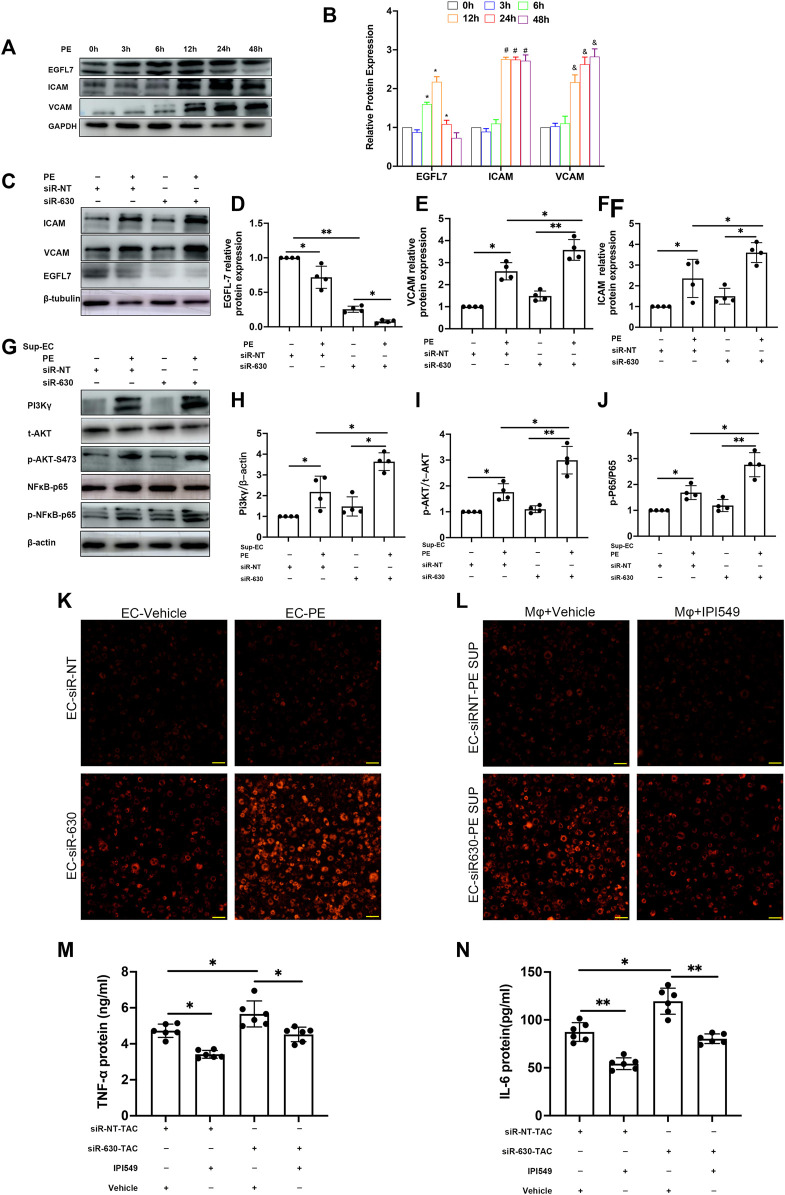
EGFL7 KD upregulated adhesion cytokines in ECs and activated macrophages through PI3Kγ/AKT/NFκB signaling. **(A)** MAEC was stimulated by phenylephrine (PE, 100 μM) for multiple time points. representative immunoblots of EGFL7, ICAM, VCAM, and quantitative analysis **(B)**, these proteins’ relative expression was normalized to GAPDH (*p < 0.05 vs. 0 h in EGFL7, #*p* < 0.05 vs. 0 h in ICAM, and *p* < 0.05 vs. 0 h in VCAM by one-way ANOVA followed by Tukey’s test). **(C)** MAEC was transfected with siRNA-630 or siRNA-NT. Representative immunoblots of EGFL7, ICAM, VCAM in MAECs treated with phenylephrine (PE,100 μM, 24 h) after transfected with siRNA-630 or siRNA-NT. **(D–F)** quantitative analysis of EGFL7, ICAM, VCAM (*p < 0.05,**p < 0.01 by one-way ANOVA followed by Tukey’s test).**(G)** representative immunoblots of PI3Kγ/AKT/ NFκ B expression in macrophages received MAECs supernatant treated with different stimulation. **(H–J)** quantitative analysis of protein expression, normalized to β-actin or t-AKT, or p65. Relative expression was calculated using ImageJ software (*p < 0.05, **p < 0.01 by one-way ANOVA followed by Tukey’s test). **(K)** representative fluorescence microscope image showed macrophages received MAECs supernatant treated with PE + siRNA-630 promoted endothelium-macrophage adhesion. scale bar = 20 μm. **(L)** representative fluorescence microscope image showed macrophages that pretreatment with PI3Kγ inhibition IPI-549 reduced endothelium-macrophage adhesion. scale bar = 20 μm. **(M, N)** quantitative analysis of TNF-α, IL-6 expression by ELISA in TAC serum treated with PI3Kγ inhibition IPI-549 or vehicles (*p < 0.05, **p < 0.01 by one-way ANOVA followed by Tukey’s test). All data represent the mean ± SD from at least 4 independent experiments.

To explore the mechanism involving the pathological process in EGFL7 KD mice, we examined the expression of EGFL7, intercellular cell adhesion molecule-1 (ICAM), and vascular cell adhesion protein-1 (VCAM) in MAECs. MAECs were stimulated with phenylephrine (PE) at a series of time points. PE stimulation for 6 h induced EGFL7 expression upregulation in MAECs (Figures 4A,B), and it peaked at 12 h post-PE. The response decreased at 24 h, although a significant difference remained. At 48 h, the expression of EGFL7 returned to the basal line. Conversely, ICAM, and VCAM showed no changes compared with the control group within 6 h. After 12 h of PE stimulation, the expression of ICAM and VCAM reached a high level (Figures 4A,B). As 12–24 h of PE stimulation led to increased EGFL7 and increased ICAM/VCAM. We decided to knock down EGFL7 at this time point to explore its mechanism.

MAECs were transfected with siRNA-630 and then stimulated with PE for 24 h. The relative expression of EGFL7 after PE stimulation was observed by western blotting (Figures 4C,D). siRNA-630 led to an approximately 80% reduction in EGFL7, and a more significant decrease was seen in the siRNA-630 + PE group. Conversely, marked upregulation of ICAM and VCAM was observed in the siRNA-630 + PE group versus the siRNA-NT + PE and siRNA-630 groups (Figures 4C,E,F).

### Epidermal Growth Factor-Like Protein 7-KD-EC Affects Endothelial-Macrophage Interactions by Activating PI3K γ -N Fκ B Signaling in Macrophages

The above results demonstrated that EGFL7 inhibits the expression of the adhesion molecules ICAM and VCAM. Next, we tried to reveal the mechanism involved in EGFL7 knockdown-induced cardiac remodeling and whether macrophages play a role in the pathological process. Macrophages were activated by lipopolysaccharide (LPS) at a 10 ng/ml concentration for 6 h. Supernatant from the MAEC medium stimulated with or without PE + + siRNA was added to the macrophage culture medium. Western blotting indicated that PI3K γ expression was upregulated after PE stimulation in the siRNA-NT group. Even more apparent upregulation in the siRNA-630 + + PE group (Figures 4G,H). Additionally, p-AKT and p-N Fκ B p65 showed a similar trend (Figures 4G,I,J). These results indicate that EGFL7 inhibits the activation of the PI3K γ -AKT-N Fκ  B signaling pathway in macrophages.

Adhesion assays showed that endothelial cell exposure to PE for 24 h could increase the adhesion of macrophages to MAECs. There was even more macrophage adhesion to MAECs in the siRNA-630 + PE group (Figure 4K, Supplementary Figure S4A). However, macrophages pretreated with a specific inhibitor of PI3K γ (IPI-549) showed reduced adhesion (Figure 4L, Supplementary Figure S4B). These results indicate that EGFL7 knockdown can promote the adhesion of macrophages to endothelial cells**.** *In vivo* research involving IPI549 reduced the expression of serum inflammatory cytokines TNF-α and IL-6 (Figures 4M,N).

### Epidermal Growth Factor-Like Protein 7 Treatment Reduces Remodeling and Apoptosis and Macrophage Infiltration in Transverse Aortic Constriction

**FIGURE 5:**
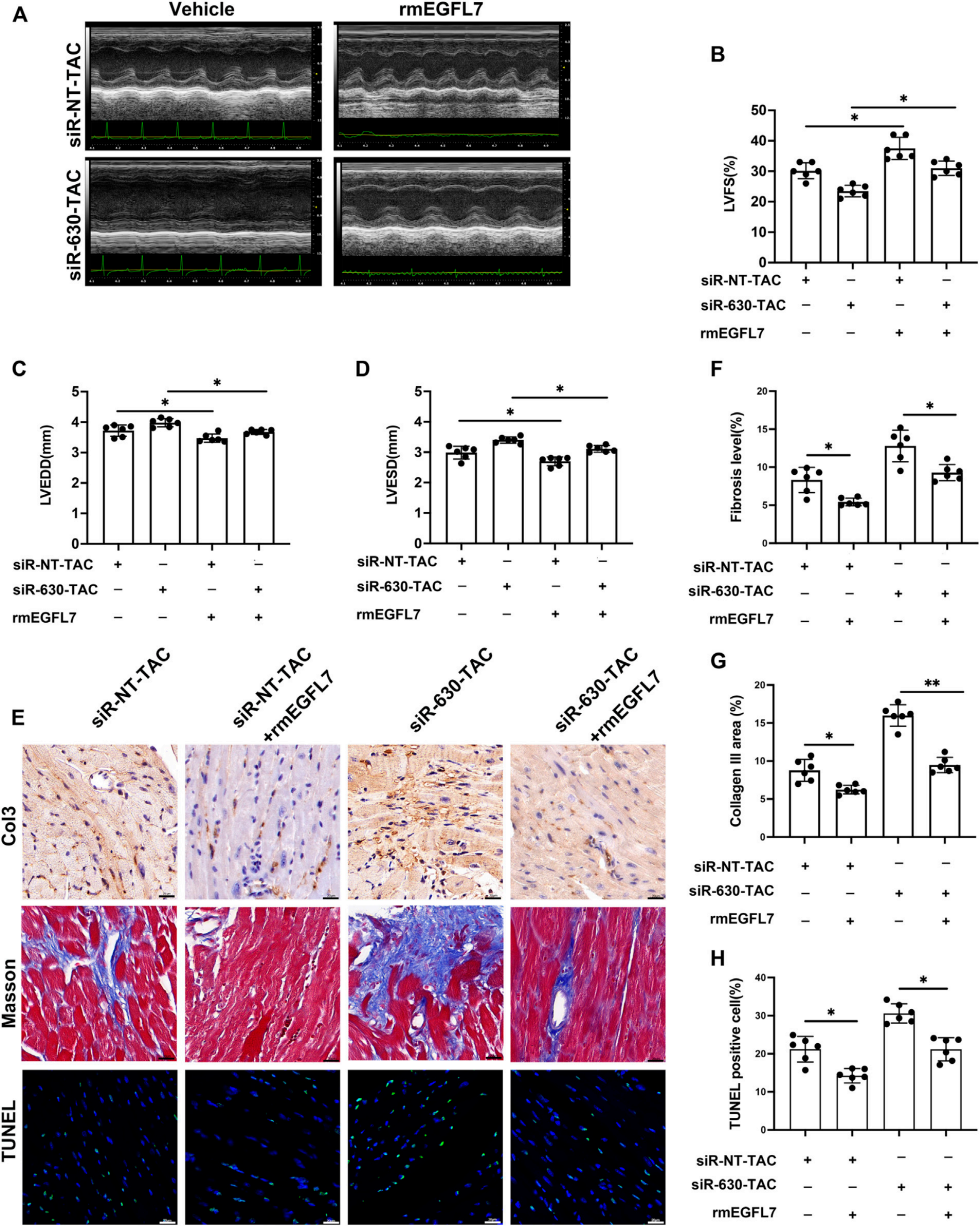
rmEGFL7 treatment reduces cardiac remodeling and apoptosis in siRNA-TAC. C57BL/6J mice were subjected to TAC operation, followed by siRNA-NT or siRNA-630 injection twice a week for 4 weeks. Recombinant mice (rm) EGFL7 or vehicles were injected intraperitoneally every day for 4 weeks. Heart function and morphological measures were observed at 4 weeks. (*n* = 6 in each group). **(A–D)** Representative echocardiograms and quantitative analysis of left ventricle fractional shortening (LVFS), left ventricle end-diastolic diameter (LVEDD), left ventricle end-systolic diameter (LVESD) (*p < 0.05, **p < 0.01 by one-way ANOVA followed by Tukey’s test). **(E, G)** Representative immunohistochemical staining of Collagen III and quantitative analysis (*p < 0.05, **p < 0.01 by one-way ANOVA followed by Tukey’s test). scale bar = 20 μm. **(E, F)** representative Masson image indicated fibrosis and quantitative analysis (*p < 0.05, **p < 0.01 by one-way ANOVA followed by Tukey’s test). Scale bar = 20 μm. **(E, H)** representative TUNEL images indicated apoptosis and quantitative analysis (*p < 0.05, **p < 0.01 by one-way ANOVA followed by Tukey’s test). Scale bar = 20 μm. All data represent the mean ± SD from at least 4 independent experiments.

Knockdown of EGFL7 in TAC mice was associated with deterioration of heart failure, thus indicating the protective role of EGFL7. We continued to explore whether supplementation with rmEGFL7 can rescue cardiac remodeling induced by its knockdown. Echocardiography demonstrated that rmEGFL7 significantly improved heart function compared with the siRNA-630-TAC group (Figures 5A–D). Immunohistochemical staining of collagen III and Masson’s trichrome staining showed a reduction in interstitial and perivascular fibrosis in the rmEGFL7 group (Figures 5E–G, Supplementary Figures S5D,E). IHC also indicated that the infiltration of CD68^+^ macrophages was significantly reduced (Supplementary Figures S5B,C). Apoptosis characterized by TUNEL also showed a significant decrease in the rmEGFL7 treatment group compared with the relative siRNA-TAC group (Figures 5E,H).

## Discussion

The present research revealed the relationship between EGFL7 and pressure overload-induced cardiac remodeling. Our results indicated that EGFL7 could suppress cardiac remodeling by inactivating macrophage inflammatory signaling. We obtained the following principal findings: 1) There was differential expression of EGFL7 through hypertrophy to heart failure. This upregulated expression in the hypertrophic stage may be compensated. 2) EGFL7 can exert its protective role through its anti-inflammatory activities. 3) The protective role of EGFL7 in pressure overload is related to endothelial cell-macrophage interactions. Our *in vitro* results indicate that EGFL7 can reduce adhesion cytokines in ECs and inhibit the PI3C γ /AKT/ NFκB signaling pathway in macrophages.

Commonly, EGFL7 protects ECs from low oxygen- or low nutrient-induced cell death (Bicker and Schmidt, 2010; Johnson et al., 2013). Some studies indicate that EGFL7 is correlated with inflammation in animal models. Badiwala et al. (2010) revealed that EGFL7 exerts protection against hypoxia/reoxygenation-induced human coronary artery endothelial cell injury by inhibiting NF κ B activation and ICAM expression (Badiwala et al., 2010). Another study (Badiwala et al., 2011) showed that EGFL7 plays a significant anti-inflammatory effect by activating Notch signaling and blocking NF κ B in calcineurin-mediated inhibition of endothelial injury. Additionally, EGFL7 can restrict CNS immune infiltration through adhesion to its ligand, integrin αvβ3, on activated T cells. Supplementation with EGFL7 can alleviate experimental autoimmune encephalomyelitis (EAE) (Larochelle et al., 2018). Delfortrie et al. (2011) found that tumors with high EGFL7 expression had fewer infiltrated immune cells and adhesion molecules. EGFL7 expression negatively correlates with tumor prognosis. Short cardiac capillaries could reduce the oxygen supply of hypertrophic hearts. It can also induce cardiomyocyte death and fibrosis. This is one of the mechanisms involved in pathological cardiac hypertrophy (Gogiraju et al., 2019). Our study first detected differential expression of EGFL7 from hypertrophy through to heart failure. The results showed that EGFL7 was upregulated at the early hypertrophic stage but returned to the basal line when heart failure progressed. Knockdown of EGFL7 by siRNA *in vivo* indicated worsened cardiac function and remodeling. Treatment with rmEGFL7 resulted in an inverse response. This meant that EGFL7 exerts protection against heart failure progression. This protective effect is similar to its anti-inflammatory effect in the central nervous system (CNS) (Larochelle et al., 2018). In the chronic stage, we detected EGFL7 expression until 8 weeks after TAC and found that EGFL7 was downregulated. In contrast, in the CNS model, EGFL7 was upregulated. We speculate that chronic pressure overload stress causes endothelial cell damage and prevents endothelial cells from secreting sufficient EGFL7 to play a protective role. Different disease models may induce differential expression.

Cardiovascular diseases are closely related to inflammation. Some research reports that some proinflammatory cytokines, including TNF-α and interleukin-6 are increased in hypertensive patients with heart failure. they are relevant to the severity of disease (Briasoulis et al., 2016; Dick and Epelman, 2016). The main mechanisms of heart failure with preserved ejection fraction (HFpEF) are oxidative stress, systemic inflammation, and endothelial dysfunction. In particular, inflammatory activation can be regarded as pre-HFpEF (Gomberg-Maitland et al., 2016). Emerging studies indicate that inflammation of the coronary microvasculature promotes the pathogenesis of HFpEF (Lim et al., 2015).

Many immune cells, such as neutrophils, macrophages, and T cells, can highly express PI3K γ . The migration of leukocytes in inflammation and immunity is closely related to PI3K γ (Rückle et al., 2006; Garcia et al., 2018). Many studies have revealed that PI3K γ is involved in inflammation-related diseases. Inhibition of PI3K γ can have a promising effect. PI3Kγ KO can alleviate inflammation-driven pulmonary fibrosis (Russo et al., 2011). PI3K γ (−/−) or pharmacological inhibition can decrease the activation and adhesion of leukocytes in graft-versus-host disease (Castor et al., 2011). In high-fat diet-induced insulin resistance, infiltration of proinflammatory macrophages increased significantly, while inflammatory and insulin resistance was suppressed in PI3K γ ^−/−^ mice (Kobayashi et al., 2011). Macrophages play an essential role in the progression of atherosclerotic plaques. Inhibition of PI3K γ function by drug or gene manipulation delays the progression of atherosclerotic plaque instability (Fougerat et al., 2008; Anzinger et al., 2012). This indicates that PI3K γ is closely correlated with inflammation.

Damilano et al. (2011) showed that PI3K γ KD mice and selective inhibitors prevent cardiac dysfunction and fibrosis. These maladaptive remodeling processes are achieved by modulating the PI3K γ activity of cardiomyocytes and macrophages (Damilano et al., 2011). TAC commonly induces an obvious pathological phenotype, including macrophage infiltration and fibrosis. Depletion of macrophages by clodronate liposomes significantly improves fibrosis and hypertrophy (Dick and Epelman, 2016; Kain et al., 2016). Our results were similar to those of previous studies. Furthermore, our results showed that depletion of macrophages rescued cardiac remodeling induced by EGFL7 siRNA. This revealed that endothelial cell-macrophage interactions might mediate cardiac remodeling under TAC.

*In vitro* studies revealed that EGFL7 has a powerful anti-inflammatory function. EGFL7 inhibits ICAM upregulation under hypoxia/reoxygenation injury. Additionally, it blocks neutrophil adhesion to HCAECs in CNI-induced endothelial dysfunction by blocking NF κ B and ICAM activation (Badiwala et al., 2010; Badiwala et al., 2011). Our results also show that ECs compensate for increasing EGFL7 to counteract ICAM and VCAM in the hypertrophic stage; nevertheless, chronic pressure overload leads to endothelial dysfunction, further resulting in less sufficient EGFL7 production. This is consistent with previous studies. Furthermore, we showed an interaction between EC-secreted EGFL7 and macrophages *in vitro*. EC supernatant of siRNA-EGFL7+PE activated macrophage PI3Kγ/AKT/NF κ B signaling and further triggered inflammation. Inhibition of PI3Kγ in macrophages with IPI549 rescued the upregulation *in vitro*. Additionally, IPI549 rescued the upregulation of TNF-α and IL-6 *in vivo*. Our results suggest that PI3Kγ/AKT/NF κ B signaling mediates phenotypic changes under TAC. Some research has indicated that αvβ3, as an EGFL7 ligand, is expressed on T lymphocytes and that EGFL7 mediates the CNS inflammatory decrease. As macrophages also express αvβ3, we will conduct relevant research.

In conclusion, we identified that EGFL7 plays an essential role in pressure overload-induced cardiac remodeling and heart failure. It can limit macrophage infiltration and decrease cardiac remodeling by inhibiting VCAM/ICAM in ECs and inactivating PI3Kγ/AKT/NF κ B signaling in macrophages.

## Material and Methods

### Human Serum Sample

Samples from control, hypertension, or hypertension combined with heart failure were obtained from patients at 960 Hospital of PLA (The General Hospital of Jinan Command). The research was approved by the institutional ethics committee of 960 Hospital of PLA (The General Hospital of Jinan Command). All participants were provided with informed written consent.

### Cell Culture and Transfection

MAEC (Otwo, HTX2423); Raw264.7 cells were cultured following manufacturers’ instructions. Three interference fragments targeted at EGFL7 and negative control, GAPDH 420 control were constructed. Eventually, siRNA-630 was selected for follow-up research. siRNA630 sequence: F: GGA​AUG​GAG​GGA​GUU​GCA​UTT, R: AUG​CAA​CUC​CCU​CCA​UUC​CTT. Lipofectamine TM 2000 reagent (lipo 2000, Invitrogen) was used to transfect siRNA-630. After 6 h, Opti-MEM reduced serum was changed to a normal medium.

### Adhesion Analysis

Macrophages adhesion to confluent MAEC monolayers on 6 well plates that have been subjected to PE with siRNA-630 was assessed with adhesion assay by Dil (cell membrane red fluorescent probe, Beyotime, C1036) according to manufacture’ instruction. Briefly, LPS activated macrophages were incubated with Dil (5 μmol/L) for 15 min at 37°C. Then macrophages were allowed to adhesion to the monolayer of MAEC for 30 min in the incubator. Then macrophages that did not adhere to MAEC would be washed off by PBS. Fluorescent macrophages were counted under a fluorescence microscope.

### Treatment With Methoxy Modified siRNA-Epidermal Growth Factor-Like Protein 7, PI3kγ Inhibitor, Recombinant Mouse Epidermal Growth Factor-Like Protein 7, Clodronate Liposome

Methoxy modified (2 ′-OME) siRNA-630 or siRNA-NC was constructed by Genepharma. After TAC was performed, each mouse was intraperitoneally injected with 2 nmol twice a week and continued for 4 weeks (Landen et al., 2006; Rungta et al., 2013). Entranster™ *in vivo* was used to enhance the transfection effect of siRNA (Guo et al., 2021). PI3Kγ inhibitor (Eganelisib, IPI549, Cat.1693758-51-8) was administered orally at a concentration of 15 mg/kg/day for a continuous 4 weeks. Recombinant mouse EGFL7 (rmEGFL7) 10 µg/ml (DGpepides Co., Ltd.) or vehicle was administered intraperitoneally every other day for 4 weeks (Larochelle et al., 2018). Clodronate liposome (Yeasan, 40337ES08) or Clodronate control was tail vein injected according to previous research for 4 weeks (Jiang et al., 2019).

### Immunoblot Analysis

Whole-cell lysates or mice Ventricular tissue was homogenized in RIPA buffers (P0013B, beyotime) containing protease inhibitors (PMSF36978, Thermo Scientific™), and Phosphatase inhibitors. According to manufacturers’ instructions, centrifugation was performed at 14,000 g for 5 min after full lysis. The supernatant was taken for subsequent Western. After protein concentration was measured. Equal amounts of protein were separated by SDS-PAGE and then transferred onto PVDF membranes. Blots were blocked in 1 × TBST with 5% milk or BSA before incubation overnight at 4°C with corresponding primary antibodies. Blots were washed with 1 × TBST, 1:10,000 secondary antibody conjugated with HRP was incubated at room temperature for 1 h, membranes were visualized by GE AI600 *via* chemiluminescence and were analysed with ImageJ. primary antibodies used in this study were included in (Supplementary Table S1)**.**

### Quantitative RT-PCR

Total RNA was isolated using TRIZOL reagent. RNA was reverse transcribed into cDNA with Prime Script RT Reagent Kit (Takara; RR037A). Subsequently, Q-RT-PCR was performed *via* Light Cycler 480 SYBR Green I Master (Roche; 04887352001). The expression levels of RNA were normalized to GAPDH. Primers for mouse gene expression are shown in the table (Supplementary Table S2). Relative quantitation was determined by the 2^—△△CT^ method.

### ELISA

Patients and mice mouse blood were collected in a clean test tube, coagulated at room temperature, and centrifuged at 2,000 g for 15 min. The serum was collected and stored at −80°C after dividing. The biomarkers human EGFL7 ELISA (TAE-881), human IL-6 (ML028583), human MCP-1 (ML058218), human TNF-α (ML77385) and mice TNF-α (MTA00b, Quantikine™ mouse TNF-a Immunoassay), mice IL-6 (ML002293) was quantified by ELISA kit.

### Transverse Aortic Constriction Surgery

TAC surgery was performed on anesthetized 8–12 weeks mice as described previously (Zaw et al., 2017) mice were randomly assigned to sham or TAC surgery. Briefly, the operating field was disinfected with 75% alcohol, and surgical tools were sterilized; the heating pad was maintained at 37°C ± 1°C. 2% isoflurane was used to maintain anesthetization. A 27-gauge blunt needle was used to yield a 0.4 mm narrow in diameter, 7.0 silk suture ligature was performed. 6.0 silk suture ligature was used to close the rib cage and skin. Sham-operated mice performed the same operation except for constriction of the aortic arch.

### Ultrasound Echocardiography

Ultrasound echocardiography (Vevo 2100, Visual Sonics, Toronto, Canada) with a transducer frequency of 40 HZ was used. 5% isoflurane was inhaled to induce anesthetization, and 1.5%–2% isoflurane for maintaining anesthetize during the operation. Heart rate of the mice was maintained between 450-500 beats/min. The heart structure was recorded by M-mode ultrasound at the papillary muscle section. Analysis was performed using software Vevo2100. Parameters including LVEDD, LVESD, LVEF, LVFS (%) were used to evaluate the changes in cardiac function.

### Immunohistochemistry Analysis

Ventricles were fixed in 4% paraformaldehyde (PFA) for 24 h, embedded in paraffin. Tissues were cut into sections of 5 um thickness for immunohistochemistry (IHC), hematoxylin, and eosin (H&E), for histological analysis according to manufacturers’ instruction (PV-9000 for IHC). Fibrillar collagen was detected with Masson’s trichrome (Solarbio G1340 for Masson). Subsequently, sections were blocked protein and endogenous enzymes, antigen retrieval, primary antibodies used in this study were included in Supplementary Table S1. Analysis was performed by ImageJ software for quantification of fibrosis. Sections were imaged using a bright field and were analysed for percentage collagen content and cross-sectional area by ImageJ. Six myocardial sections of each mouse were selected for histopathological analysis to calculate the area of interstitial or perivascular fibrosis (the ratio of the sum of the total area of interstitial fibrosis to the sum of total connective tissue area) (Krishnan et al., 2019).

### TUNEL

TUNEL assay was performed according to the manufacturer’s directions (TUNEL Andy Fluor TM 488 Apoptosis Kit, A050, ABP biosciences). The sections were examined using fluorescent microscopy. The number of TUNEL-positive cells was counted in 6 randomly selected fields under x400 magnification for each mouse. Six mice were studied per group.

## Data Availability

The original contributions presented in the study are included in the article/Supplementary Material, further inquiries can be directed to the corresponding authors.
