# Supplementary material for: Recombinant EGFL7 Mitigated Pressure Overload-Induced Cardiac Remodeling by Blocking PI3K γ /AKT/ NFκB Signaling in Macrophages
Source: Front Pharmacol. 2022 May 26;13:858118. doi: 10.3389/fphar.2022.858118 (PMC9200063; doi:10.3389/fphar.2022.858118)

Fig1e-EGFL7

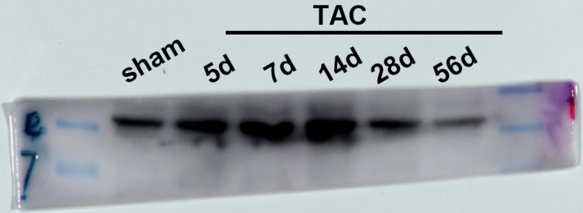

Fig1e-tubulin

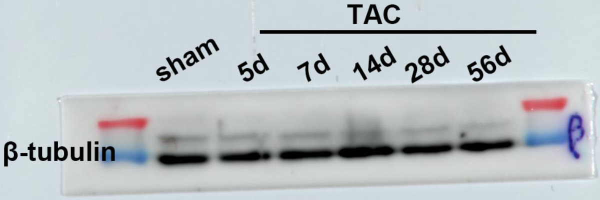

Fig4g-actin

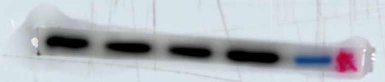

Fig4g-pP65

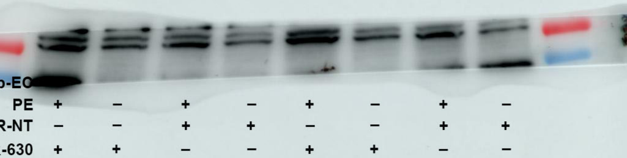

Fig4g-p65

Sup-EC

PE	+	–	+	–
siR-NT	–	–	+	+
siR-630	+	+	–	–

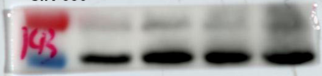

Fig4g-PI3Kγ

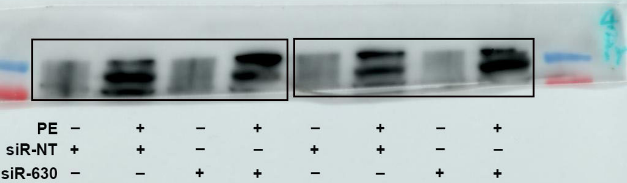

Fig4g-t-AKT

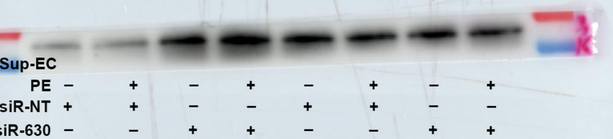

Fig4g-p-AKT

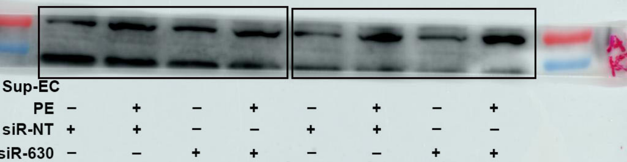

Fig4c-EGFL7

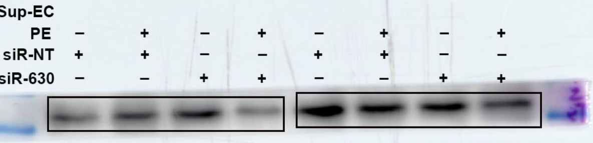

Fig4c

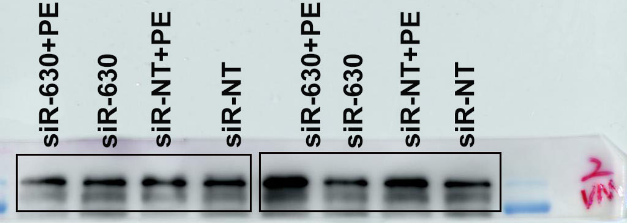
siR-630+PE
siR-630
siR-NT+PE
siR-NT
siR-630+PE
siR-630
siR-NT+PE
siR-NT

Fig4c

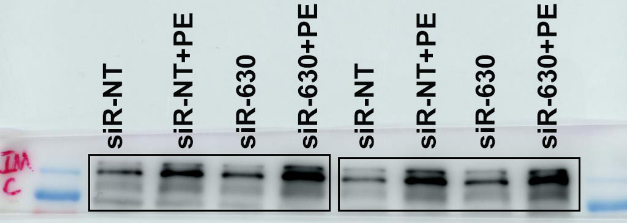
siR-NT
siR-NT+PE
siR-630
siR-630+PE
siR-NT
siR-NT+PE
siR-630
siR-630+PE

Fig4c

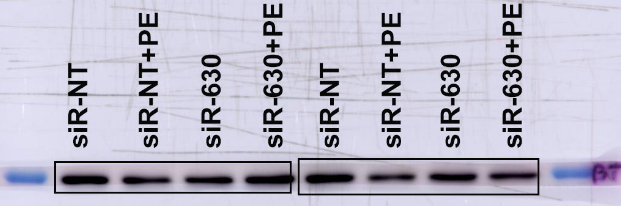
siR-NT
siR-NT+PE
siR-630
siR-630+PE
siR-NT
siR-NT+PE
siR-630
siR-630+PE

Fig4a-icam

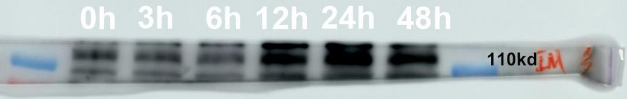

Fig4a-vcam

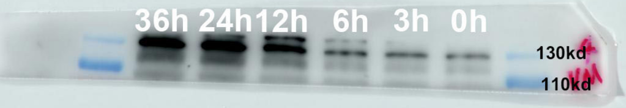

Fig4a-egfl7

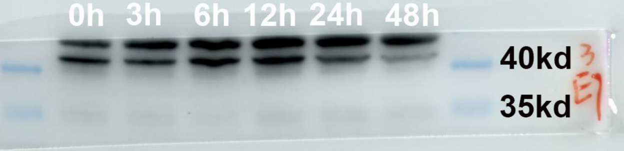

Fig4a-GAPDH

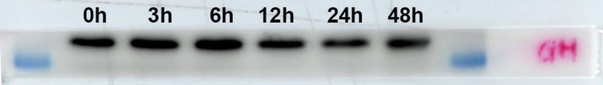

Sup.Fig1

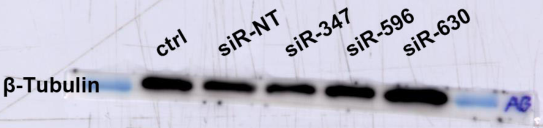

Sup.Fig1

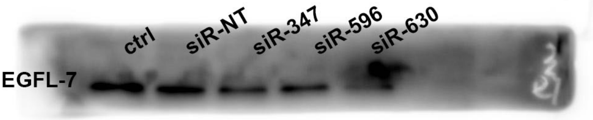

Supplement: Supplementary file 1 [file DataSheet1.PDF]
