# Supplementary material for: Recombinant EGFL7 Mitigated Pressure Overload-Induced Cardiac Remodeling by Blocking PI3K γ /AKT/ NFκB Signaling in Macrophages
Source: Front Pharmacol. 2022 May 26;13:858118. doi: 10.3389/fphar.2022.858118 (PMC9200063; doi:10.3389/fphar.2022.858118)
Supplement: Supplementary file 2 [file DataSheet2.docx]

Supplementary Material

# 1.Supplementary Figures


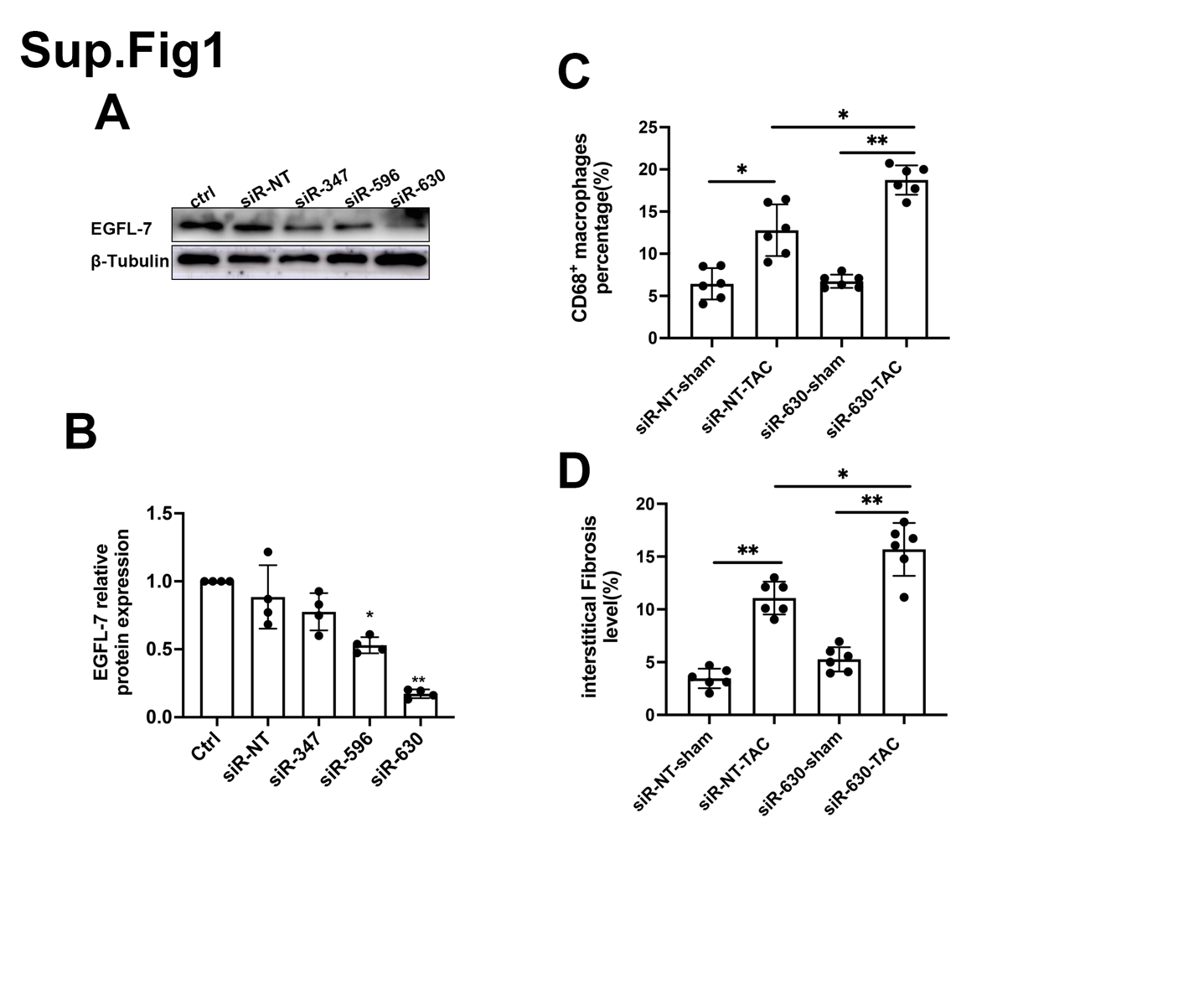


**Supplementary Figure 1. (A)** representative blotting indicated knockdown efficiency of siRNA on EGFL7and its quantitative analysis**(B)**. *P<0.05, **P<0.01vs Ctrl group by one-way ANOVA followed by Tukey’s test. **(C.D)** quantitative analysis of CD68+ macrophages percentage and interstitial fibrosis level in siRNA-NT-TAC, siRNA-630-TAC, and corresponding sham group (n=6 for each group). scale bar=20μm. (*P<0.05, **P<0.01 by one-way ANOVA followed by Tukey’s test). All data represent the mean$\pm$SD from at least 4 independent experiments.


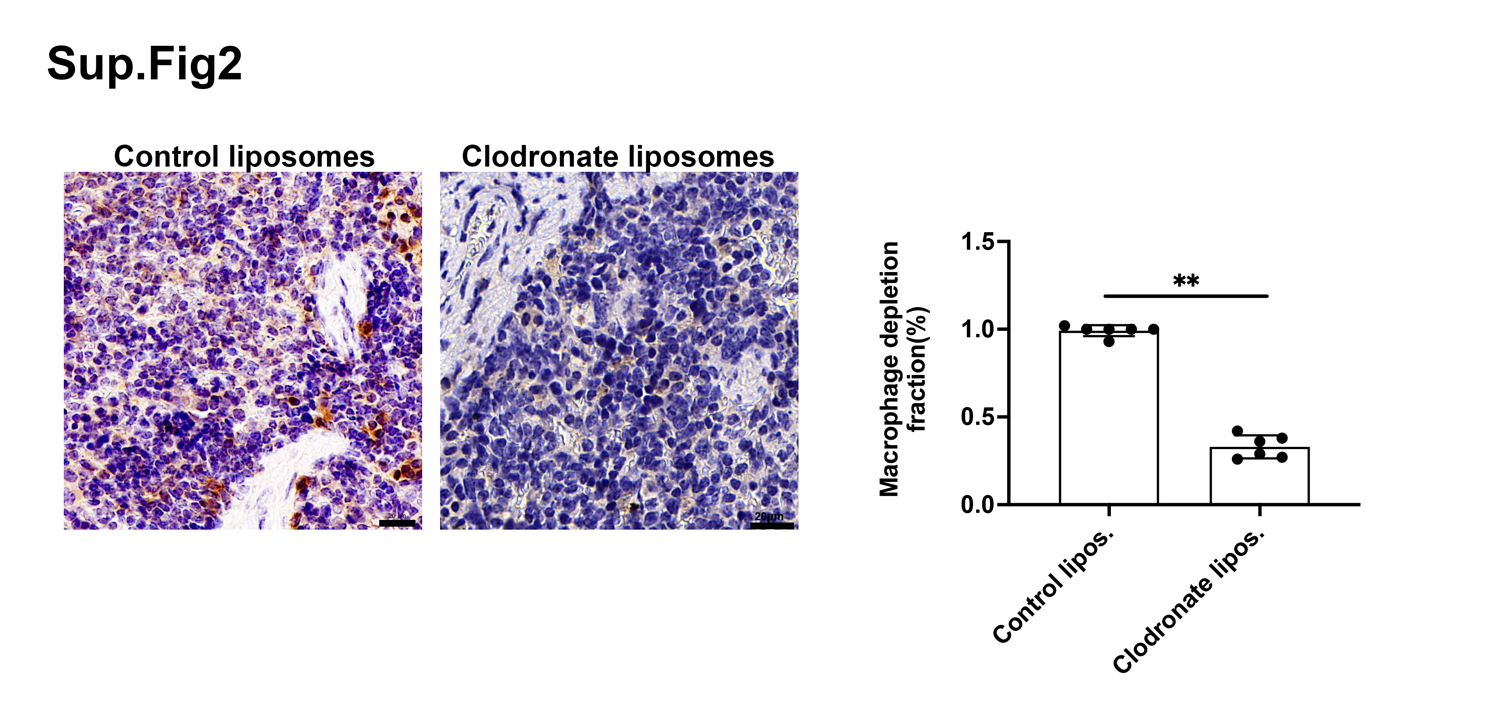


**Supplementary Figure2** Clodronate liposome or Clodronate control was tail vein injected every other day from the second day after TAC for 4 weeks. Two days after injection, deletion efficiency was valued (n=6 for each group). Representative immunohistochemical staining of CD68 indicated deletion efficiency of macrophages in the spleen and its quantitative analysis. scale bar=20μm (***P*<0.01 by one-way ANOVA followed by Tukey’s test). All data represent the mean$\pm$SD from at least 4 independent experiments.


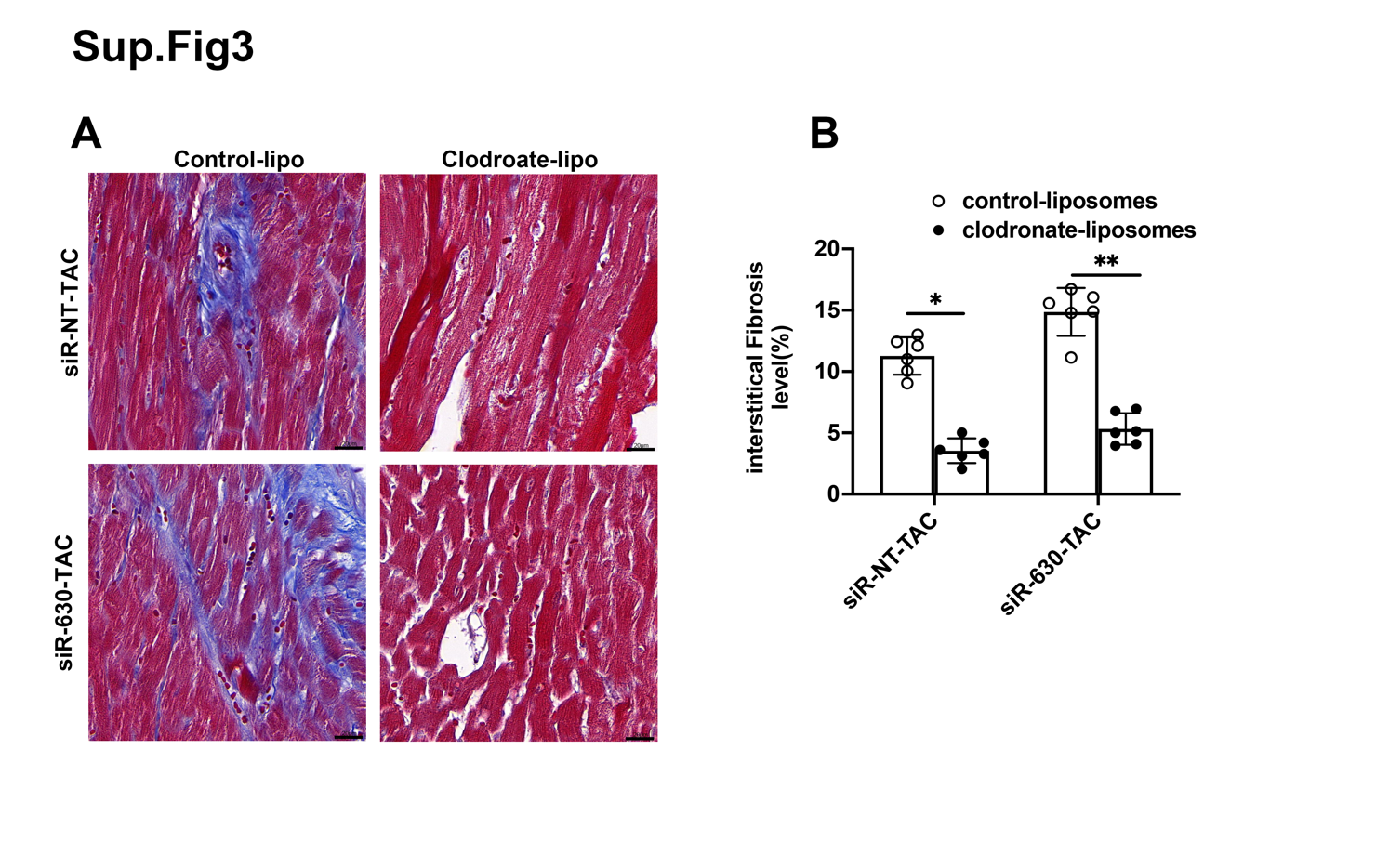


**Supplementary Figure 3**.C57BL/6J mice injected with siRNA-630 or siRNA-NT were performed TAC, and clodronate liposome or control liposome was injected for 4weeks (n=6 for each group). Representative Masson staining and its quantitative analysis indicated clodronate liposome reduced fibrosis (**P*<0.05, ***P*<0.01 by one-way ANOVA followed by Tukey’s test). All data represent the mean$\pm$SD from at least 4 independent experiments.


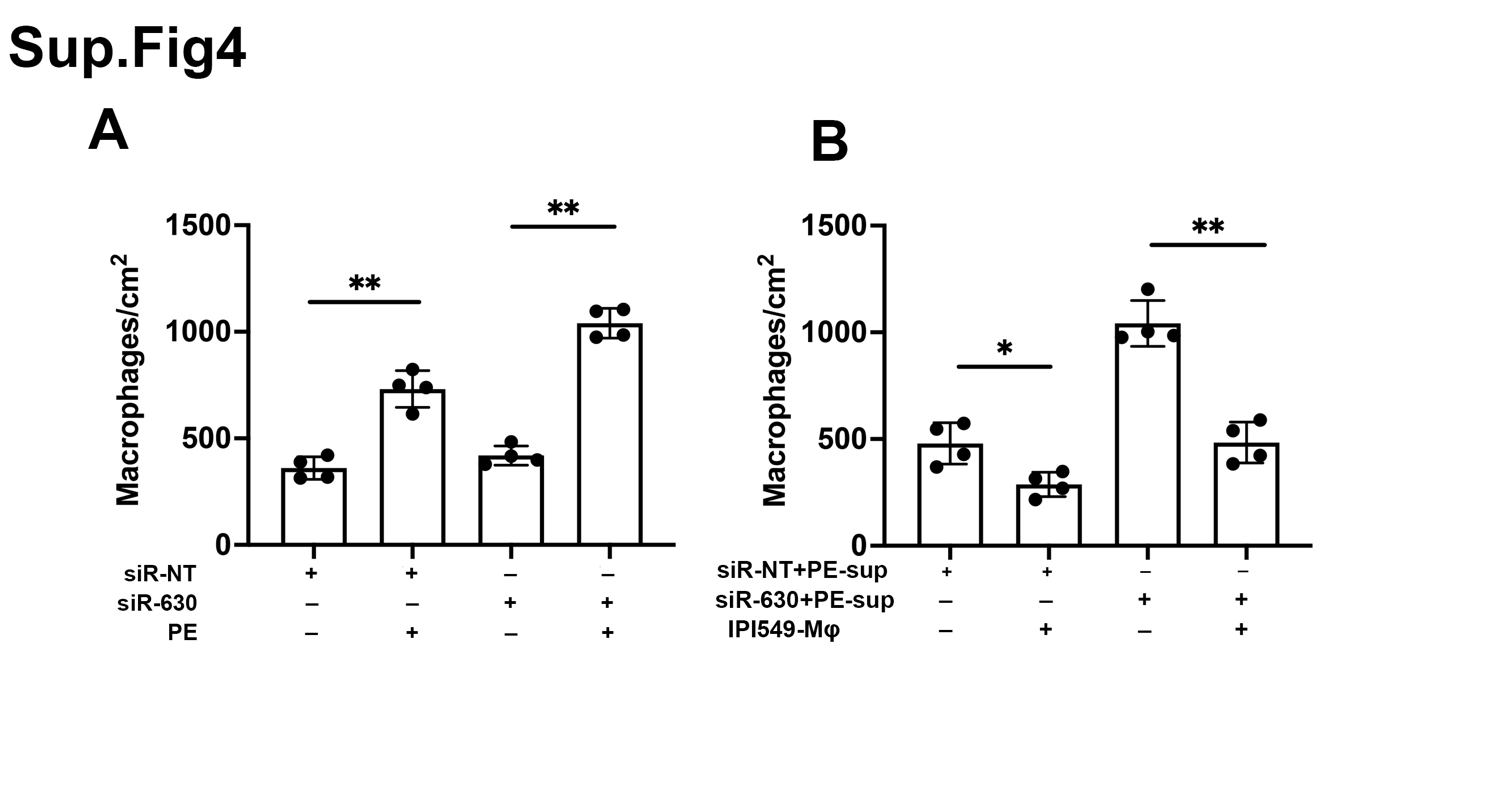


**Supplementary Figure 4** macrophages received supernatant medium from MAEC transfected with siRNA-630 or siRNA-NT with or without PE stimulation. **(A)**Adhesion assay indicates the number of macrophages adhesion with MAEC. **(B)**Activated macrophages were pretreated with PI3Kγ inhibition IPI549 or control, then received medium of MAEC. quantitative analysis indicated adhesion between macrophages and MAEC received different stimulation. (**P*<0.05, ***P*<0.01 by one-way ANOVA followed by Tukey’s test). All data represent the mean$\pm$SD from at least 4 independent experiments.


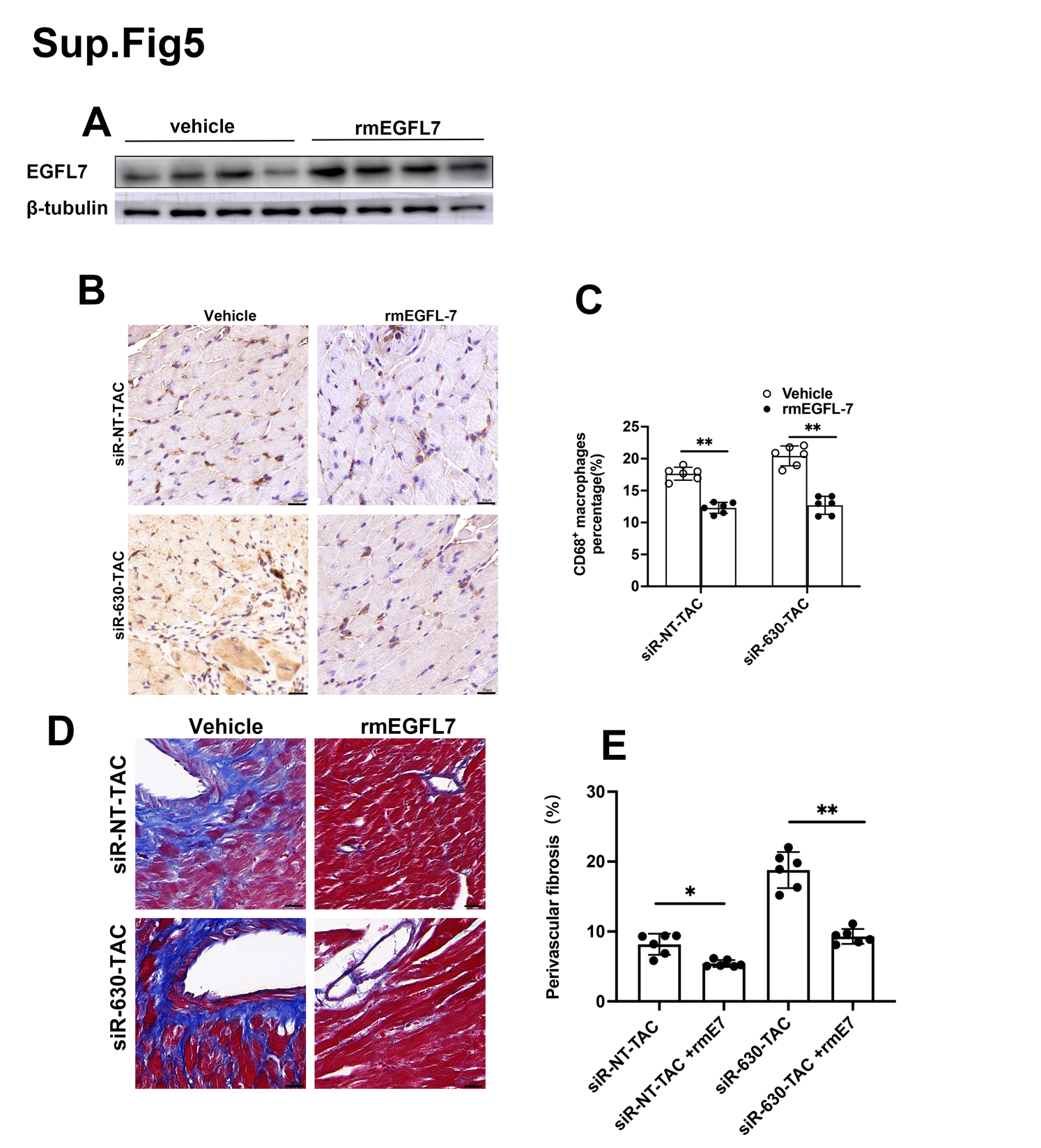


**Supplementary Figure 5**  siRNA-630 or siRNA-NT mice under TAC, then rmEGFL7 was injected for four weeks. **(A)**Immunoblots of EGFL7 in heart tissue of Vehicle or rmEGFL7 mice. **(BC)** Representative images and quantitative analysis indicated CD68^+^ macrophages decrease after EGFL7 injection**. (DE)** Representative Masson staining images indicated perivascular fibrosis and quantitative analysis after rmEGFL7 injection (**P*<0.05, ***P*<0.01 by one-way ANOVA followed by Tukey’s test).

# 2. Supplementary Tables

| **target antigen** | **source** | **catlog#** | **working concentration** |  |
| --- | --- | --- | --- | --- |
| **EGFL7** | Affinity | DF12391 | 1：500 | WB |
|  |  |  | 1：200 | IHC |
| **GAPDH** | proteintech | 10494-1-AP | 1:2000 | WB |
| **β-actin** | affinity | AF-7018 | 1:2000 | WB |
| **β-tubulin** | proteintech | 10094-1-AP | 1:2000 | WB |
| **PI3Kγ** | CST | #5405 | 1:1000 | WB |
| **AKT** | proteintech | 10176-2-AP | 1:1000 | WB |
| **p-AKT S473** | proteintech | 66444-1-AP | 1:500 | WB |
| **ICAM** | PRoteintech | 10020-1-AP | 1:1000 | WB |
| **VCAM** | boster | BA0406 | 1:1000 | WB |
| **NFκB-P65** | abcam | ab16502 | 1:1000 | WB |
| **p-P65** | abcam | ab76302 | 1:500 | WB |
| **Col3** | abcam | ab59436 | 1:200 | IHC |
| **CD68** | proteintech | 20536-1-AP | 1:200 | IHC |
| **TGF-β** | Proteintech | 21898-1-AP | 1:200 | IHC |

**Sup.Tab1** antibodies in this research.

**Sup.Tab2** primers in this research.

| **Primers** |  | **5'--3'** |  |  |
| --- | --- | --- | --- | --- |
| IL-6 | F | GCCTTCTTGGGACTGATGCT | | |
|  | R | GCCATTGCACAACTCTTTTCTCA | | |
| TNF-α | F | CGGGCAGGTCTAATTTGGAG | | |
|  | R | ACCCTGAGCCATAATCCCCT | | |
| CCL2 | F | ACCACCTCAAGCACTTCTGT | | |
|  | R | TAAGGCATCACAGTCCGAGT | | |
| CCL3 | F | CAGCGAGTACCAGTCCCTTT | | |
|  | R | GCAGTGGTGGAGACCTTCAT | | |
| MIP1a | F | CAGCGAGTACCAGTCCCTTT | | |
|  | R | GCAGTGGTGGAGACCCTTCAT | | |
| CTGF | F | AGAACTGTGTACGGAGCGTG | | |
|  | R | TGGCACCATCTTTGGCAGTG | | |
| CXCL1 | F | GGCTGGGATTCACCTCAAGAA | | |
|  | R | TGAGTGTGGCTATGACTTCGG | | |
| CXCL2 | F | CTGCCAAGGGTTGACTTCAAGA | | |
|  | R | CTTCAGGGTCAAGGCAAACT | | |
| CXCL5 | F | CFFTTCCATCTCGCCATTCA | | |
|  | R | GCTATGACTGAGGAAGGGGC | | |
| CXCL8 | F | CTAGGCATCTTCGTCCGTCC | | |
|  | R | TTGGGCCAACAGTAGCCTTC | | |
| EGFL7 | F | AGCCTTACCTCACCACTTGC | | |
|  | R | TGGCATATTGCTGCTCCACC | | |
| GAPDH | F | CCCTTAAGAGGGATGCTGCC | | |
|  | R | ACTGTGCCGTTGAATTTGCC | | |
|  |  |  | | |

**Sup.Tab3.** physiological and Echocardiographic parameters of siRNA-NT, siRNA-630 mice at baseline

|  | **siRNA-NT(n=13)** | **siRNA-630(n=12)** |
| --- | --- | --- |
| BW（g） | 25.3±0.3 | 24.9±0.43 |
| HW(mg) | 110.2±1.4 | 109.7±1.8 |
| LVW(mg) | 78.4±0.8 | 79.6±1.5 |
| Lung weight | 139.2±2.1 | 134.6±2.5 |
| Liver weight | 1057.8±36.9 | 1046.3±21.7 |
| LVW/BW | 3.13±0.06 | 3.16±0.05 |
| LVIDd | 2.75±0.02 | 2.69±0.03 |
| LVIDs | 1.46±0.02 | 1.43±0.01 |
| LVFS | 48.7±0.68 | 47.4±0.52 |

**Sup.Table3** Physiological and echocardiographic parameters of siRNA-NT, siRNA-630 mice at baseline.BW, body weight; HW, heart weight; LVW, left ventricle weight; LVIDd, LV internal diameter at end-systole; LVFS, LV fractional shortening. All data represent the mean±SD analysed by unpaired t test.

**Sup.Tab4.** physiological and Echocardiographic parameters of siRNA-NT-Sham, siRNA-NT-TAC, siRNA-630-Sham and siRNA-630-TAC mice at 4 weeks after TAC (n=6 for each group

|  | | siR-NT-Sham | | siRNA-630-Sham | | siRNA-NT-TAC | | siRNA-630-TAC | |
| --- | --- | --- | --- | --- | --- | --- | --- | --- | --- |
| LVEDD(mm) | | 3.46±0.02 | | 3.49±0.03 | | 3.87±0.05 ** | | 4.26±0.07* # | |
| LVESD(mm) | | 1.88±0.02 | | 1.9±0.02 | | 2.44±0.06 ** | | 3.11±0.1 *# | |
| LVFS% | | 45.6±0.72 | | 45.4±0.68 | | 37±0.97 ** | | 27.1±1.14 *# | |
| LVEDP(mmHg) | | 4.62±0.39 | | 5.3±0.41 | | 10.3±0.76 * | | 17.7±0.73*# | |
| dP/dtmax(mmHg/s) | | 7578±483 | | 7758±451 | | 5958±402 * | | 5123±498 *# | |
| dP/dtmin(mmHg/s) | | 7414±649 | | 7085±569 | | 5339±529 * | | 4962±573* | |
| HW | | 110.2±1.4 | | 109.7±1.8 | | 237.8±6.6^**^ | | 261.7±7.9^*##^ | |
| Lung weight | | 139.2±2.1 | | 131.6±2.5 | | 218.6±3.7^*^ | | 223.7±4.1^*#^ | |
| Liver weight | | 1052.8±36.9 | | 1043.3±21.2 | | 1569.9±46.2^*^ | | 1596.8±37.1^*#^ | |
| IVSd(mm) | 0.72±0.03 | | 0.74±0.02 | | 0.98±0.01 | | 0.92±0.02 | |  |
| LVPWd(mm) | 0.93±0.01 | | 0.94±0.02 | | 1.03±0.02^*^ | | 1.19±0.02*# | |  |
| HR（BMP） | | 749±16 | | 737±14 | | 758±14 | | 659±17*# | |

**Supplemental Figurer4** Echocardiographic parameters of siRNA-NT-Sham, siRNA-NT-TAC, siRNA-630-Sham, siRNA-630-TAC. LVEDD, Left Ventricular End Diastolic Diameter; LVESD, Left Ventricular End Systolic Diameter; LVFS, fractional shortening; dP/dtmax, peak instantaneous rate of left ventricular pressure; dP/dtmin, peak instantaneous rate of decline in left ventricular pressure increase; LVEDP, LV end-diastolic pressure. HW, heart weight; IVSd, diastolic interventricular septum wall thickness; LVPWd, diastolic left ventricle posterior wall thickness. All data represent the mean$\pm$SD; *P <0.05，**P <0.01vs corresponding sham group; #: P<0.05，##: P<0.01 vs siRNA-NT-TAC group by one-way ANOVA
